# Impact of polymyxin B hemoperfusion therapy on high endotoxin activity level patients after successful infection source control: a prospective cohort study

**DOI:** 10.1038/s41598-021-03055-8

**Published:** 2021-12-16

**Authors:** Won Young Lee, Hee Ju Kim, Eun Young Kim

**Affiliations:** 1 Department of Thoracic and Cardiovascular Surgery, Seoul St. Mary’s Hospital, Catholic University of Korea, Seoul, South Korea; 2 Department of Surgery, Seoul St. Mary’s Hospital, Seoul, Korea; 3 Division of Trauma and Surgical Critical Care, Department of Surgery, Seoul St. Mary’s Hospital, College of Medicine, The Catholic University of Korea, Banpo-daero 222, Seocho-gu, Seoul, 137-701 South Korea

**Keywords:** Medical research, Risk factors

## Abstract

We sought to evaluate the clinical implication of endotoxin levels in gram-negative bacilli (GNB)-induced abdominal septic shock patients with polymyxin B-hemoperfusion (PMX-HP) treatment. A prospective cohort of 60 patients who received surgical infectious source control for abdominal sepsis from January 2019 to December 2020 was included in the study. Endotoxin activity (EA) levels and Sequential Organ Failure Assessment (SOFA) scores were assessed immediately after surgery (baseline), 24, and 48 h post baseline. With receiver operating characteristic curves, the patients were stratified into two groups by the EA cut-off value (high-risk group vs low-risk group) and the clinical outcomes were compared. Logistic regression was performed to identify the clinical impact of PMX-HP on in-hospital death. Among the 31 high-risk patients (EA level ≥ 0.54), 16 patients (51.6%) received PMX-HP treatment and showed significant decreases in EA levels compared to patients who underwent conventional treatment only (− 0.34 vs − 0.12, *p* = 0.01). SOFA scores also showed significant improvement with PMX-HP treatment (12.8–8.9, *p* = 0.007). Fourteen in-hospital deaths occurred (45.2%), and PMX-HP treatment had a protective effect on in-hospital death (odds ratio (OR) 0.04, *p* = 0.03). In 29 low-risk patients (EA level < 0.54), seven patients (24.1%) received PMX-HP treatment and showed significant decreases in EA levels (0.46–0.16, *p* = 0.018). However, SOFA scores and in-hospital deaths were not improved by PMX-HP treatment. EA level significantly decreased after PMX-HP treatment and it may represent a therapeutic option to improve organ impairment and in-hospital death in septic shock patients with EA levels exceeding 0.54.

## Introduction

Endotoxin, which is a major component of the outer membrane of GNB, would be a powerful causative agent of systemic inflammatory response syndrome by releasing various inflammatory cytokines^1–6^. Previous studies reported worse clinical outcomes in critically ill patients with high levels of endotoxemia, especially in the case of sepsis or septic shock by progressive organ dysfunction and higher mortality compared to patients with low levels of endotoxemia^1,2,7^. Also, endotoxin activity (EA) is regarded as a useful assessment tool in evaluating the treatment response to initial sepsis management or the detection of a newly developed infection. Therefore, changes in EA levels might be feasible to not only evaluate the effect of treatment but also help to improve clinical outcomes by the early detection of new infectious complications. However, there have been only few reports on the relationship between changes in endotoxin levels after surgical infection control and the prognosis of patients with abdominal sepsis. In 2019, we conducted a comparative study of polymyxin B-hemoperfusion (PMX-HP), a hemoperfusion treatment that removes serum endotoxin, in patients with severe intra-abdominal infections compared to the control group without PMX-HP^8^. In that study, PMX-HP had a significant effect on shortening the length of intensive care unit (ICU) stay. However, we failed to identify the relationship between endotoxin levels and other clinical outcomes because PMX-HP was not assigned by actual serum EA levels due to the lack of laboratory data on EA levels.

Herein, we investigated EA levels before and after PMX-HP and their relevance to the clinical prognosis of patients who received surgery due to abdominal sepsis. We also sought to identify the appropriate target of EA in patients with abdominal sepsis who might benefit the most from PMX-HP after surgery.

## Materials and methods

### Study design and participants

This prospective cohort study was approved by the Institutional Review Board of Seoul St. Mary’s hospital (No. IRB; KC18OESI0835). From January 2019 to December 2020, excluding patients less than 18 years old, patients who were diagnosed with sepsis or septic shock due to intra-abdominal infections by ileus, bowel obstruction, or perforation were eligible for the study. Informed consent was obtained from all patients or the patient’s relative or surrogate. Among them, patients who admitted to the surgical ICU after the successful control of the infectious source through major abdominal surgery were included for analysis. Sepsis or septic shock was diagnosed according to the definition in the Surviving Sepsis Campaign guidelines and received standardized treatment according to the guidelines^9^. In addition, bacterial and fungal culturing was performed from multiple sites including the blood, sputum, urine, and surgical drain, prior to the administration of antibiotics. Empiric antibiotics was used based on our institution’s policy and the operative findings, which were mostly to treat intra-abdominal infection by meropenem or piperacillin/tazobactam, and if there was a suspicion of gram-positive infection, we consider to add vancomycin. A combination of metronidazole with quinolone, ceftazidime, or cefepime were also used. After confirming the culture results, we reassessed the antibiotic therapy according to the pathogen strain. The definition of source control in this study was the elimination of ongoing infection and the restoration of anatomical destruction involving intra abdominal organs^10^. Successful control of infection source was defined as described in Solomkin et al.; when the patient achieved these results after an invasive surgery or procedure to remove an infectious agent, (1) improvement in inflammatory levels [white blood cell (WBC) < 12,000 µL/L], (2) loss of fever [oral temperature < 37.5 °C], (3) normalization of enteric function and no further surgery or other intervention require, (4) improvement of physical findings of rigidity and tendernss^11^. Patients who refused any active resuscitation treatment were excluded from the analysis.

### Measurement of endotoxin activity and data collection

The endotoxin activity assay was conducted according to the method described by Romaschin et al., using a murine immunoglobulin M monoclonal antibody against the lipid A of *Escherichia coli* J5^12^. For each participant, the study enrollment time was since the patient admitted to surgical ICU, and a 2 ml sample of whole blood was collected into an EDTA tube at study inclusion (T0, baseline) and 24 (T24), and 48 (T48) hours after surgery. The blood samples were maintained at 18–25 °C and assayed within one hour of collection. Briefly, the LPS/anti-LPS complex primes the patient’s neutrophils for an augmented response to stimulation with zymosan. EA was measured on a scale from 0 to 1, by opsonized zymosan using chemiluminescence (Autolumat LB 953; E. G. & G. Berthold, Wildbad, Germany). Based on the results obtained, the samples were considered appropriate if the coefficient of variation between the duplicates was lower than 15% and 30% for EA levels below and above 0.2, respectively. Patients receiving immunosuppressive agents or who shows severe leukocytopenia (leucocyte count of < 500 µl/L) were excluded since endotoxin activity assay is involved by the neutrophil signal transduction pathways^12^. Demographics and clinical outcomes were prospectively collected from vitals chart and medical records from the time of study enrolment. The degree of organ dysfunction was assessed using Sequential Organ Failure Assessment (SOFA) score^13^.

### Polymyxin B-hemoperfusion for abdominal septic shock patients

Since 2016, our institution has implemented PMX-HP for selected cases after conducting source control such as surgery or surgery plus intervention for patients diagnosed with peritonitis and septic shock in the surgical ICU. According to the EUPHAS 1 trial^14^, we applied PMX-HP according to our own guidelines. The indications and contraindications for PMX-HP are listed in Supplementary Table S1. All patients adhered to the Surviving Sepsis Campaign guidelines before PMX-HP treatment, and PMX-HP was considered as a complementary therapy especially for severe patients who did not show significant resuscitation response after an initial application of the sepsis bundles.

The first PMX-HP cycle was started within 12 h after source control and the second PMX-HP cycle continued within 24 h after the first session was completed. For PMX-HP, access was obtained by inserting a dual lumen catheter (12Fr Arrow International, Reading, PA, USA) into the femoral vein or internal jugular vein under ultrasound guidance. Subsequently, two cycles of PMX-HP were performed using Toraymyxin cartridge (Toray Industries, Tokyo, Japan) in continuous renal replacement therapy (CRRT) machine. The blood flow velocity was changed to 80–120 mL/min, and as a circuit anticoagulant, Nafamostat mesylate (Futhan, Torii Pharmaceuticals, Tokyo, Japan, Japan) was used at a dose of 20–30 mg/h^15^. Each cycle was performed for six hours based on the theory of Kawazoe et al*.* except for the cases that met PMX-HP therapy discontinuation criteria^16^. With a requirement of renal replacement therapy, two CRRT machines were set and each machine mounted cartridges for PMX-HP or renal replacement therapy to perform simultaneous therapy without direct interactions.

### Statistical analysis

The categorical variables were analyzed using the χ^2^ test or Fisher’s exact test. The continuous variables were analyzed using the Student’s t-test. Normal distribution of the variables was tested using the Kolmogorov–Smirnov test, and in the case of variables that were not normally distributed, a nonparametric test was performed using the Mann–Whitney test. The Wilcoxon rank-sum test was used to analyze the paired data. A *p-*value of < 0.05 was considered statistically significant. Receiver operating characteristic (ROC) analysis was performed to determine the cutoff EA level at baseline (T0) for detecting septic shock. All participants were divided into two groups according to the EA cut-off level (high risk group vs low risk group), and variations in SOFA scores measured at each time (T0, T24, T48, and T72) were analyzed in each group classified according to PMX-HP treatment. Paired t-test or Wilcoxon signed rank test was performed to evaluate effect of PMX-HP by mean difference of EA level and SOFA score within the subgroup. To examine the PMX-HP effects on changes of EA levels over time and assess the time-by-group interaction, two-way repeated measures ANOVA was conducted after EA values were log transformed.

Logistic regression analysis was performed to identify the predictors of in-hospital death, and an additional non-lin ear effect was used to evaluate the probability of in-hospital death stratified by EA levels. Variables with a *p*-value of less than 0.2 in the univariate analyses were input into the multivariate model, in which a *p*-value of less than 0.05 was considered statistically significant. All statistical analyses were conducted using R version 4.0.3 (R Foundation for Statistical Computing, Vienna, Austria, https://www.R-project.org/)^17^.

### Informed consent statement

Informed consent was obtained from all patients or the patient’s relative or surrogate, and the procedures complied with Declaration of Helsinki.

## Results

### Patient characteristics

From January 2019 to December 2020, 60 patients were enrolled and all participants were monitored until hospital discharge. Thirty-one males were included (51.7%) and the mean age of the enrollees was 66.7 years. The median Acute physiology and Chronic Health Evaluation (APACHE) II score at ICU admission was 12.0 and the median initial SOFA score was 6.0. The most common type of surgery was bowel resection (55 cases, 91.7%) followed by vascular surgeries (4 cases, 6.7%). A total of 20 patients (33.3%) underwent cancer-related surgery. Sepsis and septic shock were diagnosed in 17 patients (28.3%) and 43 patients (71.7%), respectively, and PMX-HP was performed in 23 patients (38.3%) without any complication including circuit clotting. A total of 49 patients (81.7%) demonstrated positive microorganism culture, but there was no significant difference in the prevalence of gram-negative bacteria, gram-positive bacteria or fungi between two groups. The empirical antibiotic agents were used according to our institution’s policy. We assumed the two patients with fungal infection was not covered with the appropriate empiric agent, however, we added the antifungal agent as soon as the culture results were reported. There were 18 patients of who revealed no growth (18/49, 36.7%) in microorganism culture, which rate was comparable to both general infectious ICU patients (36.4%)^18^ and to a similar subset of infectious patients (36.7% vs 18.8–32.9%)^14,19^**.**

**Figure 1:**
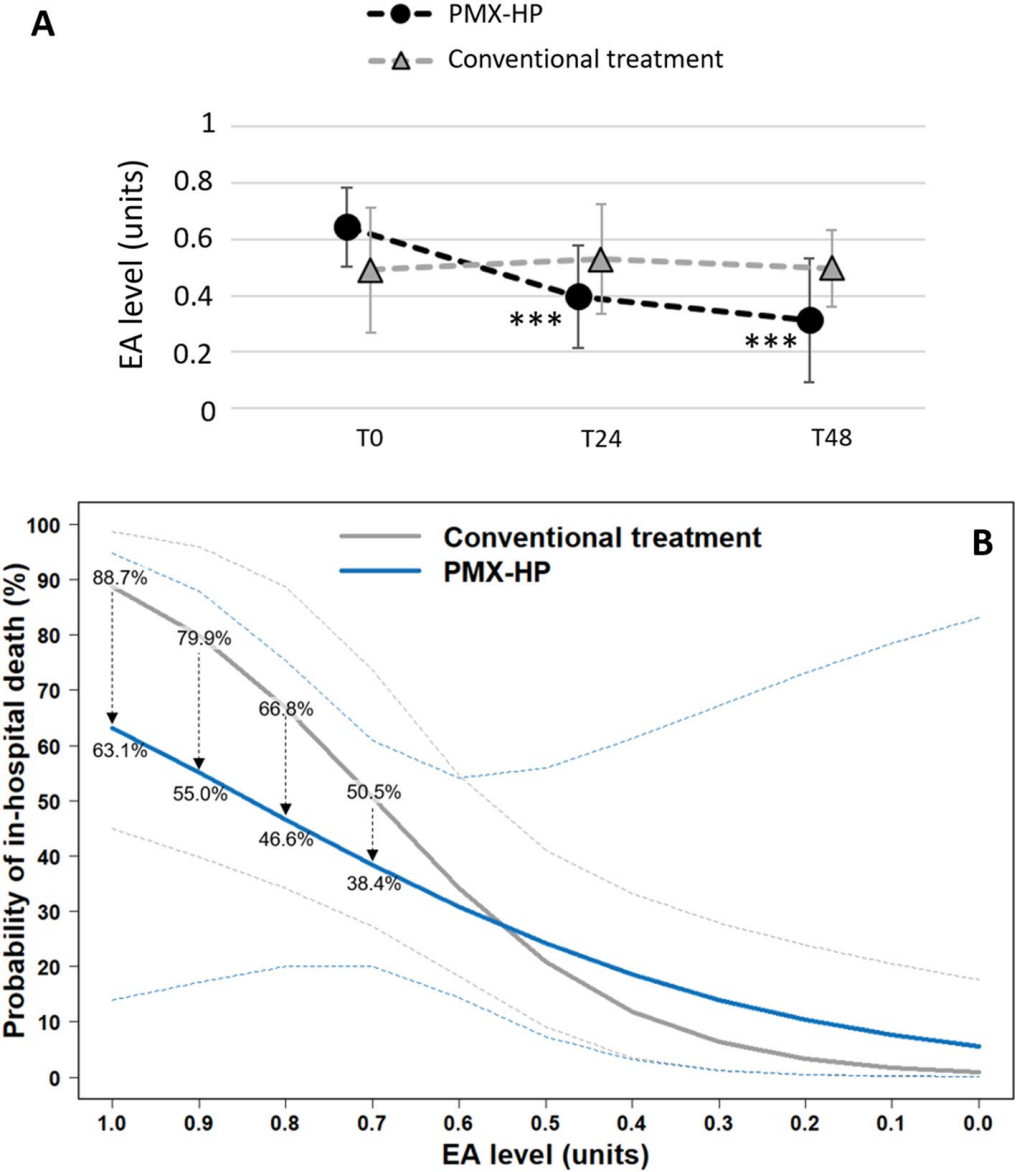
(**A**) Changes of mean (± SD) endotoxin activity levels with (black) and without (gray) PMX-HP treatment. (**B**) Probability of in-hospital death according to EA level at T0 categorized by treatment strategy. Dashed lines enclose the 95% CI. *p < 0.05 vs. baseline (T0); **p < 0.01 vs. baseline (T0); ***p < 0.001 vs. baseline (T0). *EA* endotoxin activity, *PMX-HP* polymyxin B-hemoperfusion, *SD* standard deviation.

As shown in Fig. 1-A, EA levels measured at T0, T24, and T48 showed different patterns according to whether or not PMX-HP was performed. In patients who received PMX-HP, the EA levels significantly decreased from 0.64 to 0.31 within 48 h after surgery (*p* < 0.001). In patients who underwent conventional treatment only, EA levels showed no decrease within 48 h after surgery (0.49–0.50). The two-way repeated measures ANOVA analyses showed significant time-by-group interaction effects for EA levels (F = 20.4, *p* < 0.001). Logistic regression analysis of all participants showed a significant association between EA levels at baseline (T0) and in-hospital death with an odds ratio (OR) of 1.49 (95% confidence interval [CI] 1.07–2.08; *p* = 0.018). However, PMX-HP failed to show a survival benefit (OR: 1.44, *p* = 0.53). When plotting the probability of in-hospital death stratified by the EA level at baseline (T0), and comparing the patients who received PMX-HP or not, the two curves appeared to cross at EA levels between 0.5 and 0.6 (Fig. 1-B).

**Figure 2:**
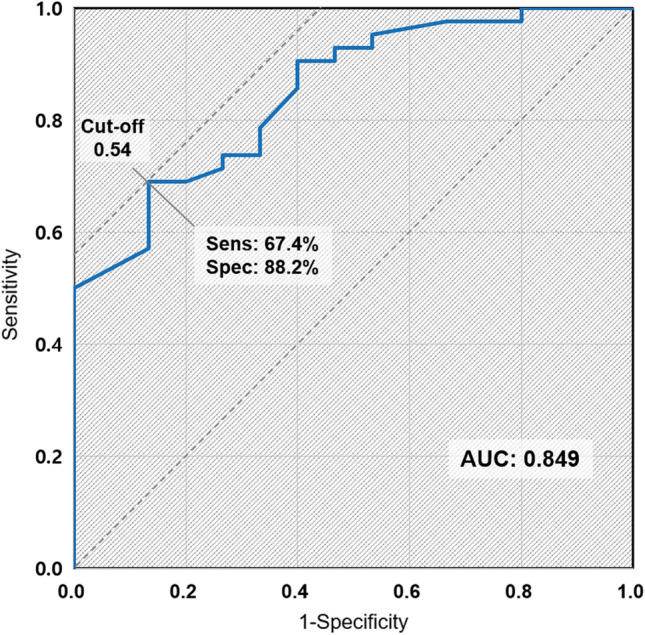
ROC curve analysis for endotoxtin activity level with measured areas under curve (AUC) of 0.849. *ROC* receiver operating characteristics.

To identify the appropriate target patients who might benefit the most from PMX-HP, we generated ROC curves to determine the EA cutoff level at baseline for the detection of septic shock, as presented in Fig. 2. The area under the curve (AUC) was 0.849 (95% CI 0.744–0.946, *p* < 0.001) and the cutoff value was 0.54. The patients were divided into two groups based on the cutoff level where an EA level of ≥ 0.54 was the high-risk group (n = 31, 56.7%) and an EA level of < 0.54 was the low-risk group (n = 29, 48.3%). The results of the comparative analysis of the baseline characteristics between the two groups are summarized in Table 1. The high EA group experienced more clinical severity by higher SOFA scores (10.0 vs 2.0, *p* < 0.001), higher APACHE II scores (19.0 vs 9.5, *p* < 0.001), higher lactate level (4.6 vs 2.2, *p* = 0.006), and more frequent septic shock (93.5% vs 48.3%, *p* < 0.001). Worse clinical outcomes were also identified with the in-hospital mortality rate (45.2% vs 13.8%, *p* = 0.01).

**Table 1:** Baseline characteristics and clinical outcomes of patients subdivided for EA levels measured at baseline (T0).

	All patients (n = 60)	High EA at T0 (EA ≥ 0.54, n = 31)	Low EA at T0 (EA < 0.54, n = 29)	*p* value
**Baseline characteristics**				
Age, years (median, IQR)	68.5 [59.6–77.0]	71.0 [60.0–78.0]	67.0 [57.0–74.0]	0.39
Sex, Male, n (%)	31 (51.7%)	16 (51.6%)	15 (51.7%)	0.99
Underlying malignancy, n (%)	26 (43.3%)	13 (41.9%)	13 (44.8%)	0.82
Disease severity				
SOFA score (median, IQR)	6.0 [2.0–12.0]	9.0 [6.8–15.3]	2.0 [1.3–4.8]	< *0.001*
APACHE II (median, IQR)	12.0 [8.0–22.5]	19.0 [12.0–30.3]	9.5 [6.0–11.0]	< *0.001*
Indications for surgery, n (%)				
Bowel perforation	36 (60.0%)	23 (74.2%)	13 (44.8%)	*0.02*
Cancer-related problem	13 (21.7%)	2 (6.5%)	11 (37.9%)	*0.004*
Bowel ischemia	7 (11.7%)	5 (16.1%)	2 (6.9%)	0.43
Others	4 (6.7%)	1 (3.2%)	3 (10.3%)	0.35
Duration of surgery, min (mean, ± SD)	199 ± 104	178 ± 83	220 ± 120	0.13
Type of isolated microorganism				
No. of samples available	49 (81.7%)	26 (84.9%)	23 (79.3%)	0.65
No growth	18 (36.7%)	5 (19.2%)	13 (56.5%)	*0.007*
Gram negative	9 (18.4%)	5 (19.2%)	4 (17.4%)	> 0.99
Gram positive	8 (16.3%)	7 (26.9%)	1 (4.3%)	0.052
Fungi	2 (4.1%)	1 (3.8%)	1 (4.3%)	> 0.99
Mixed	12 (24.5%)	8 (30.8%)	4 (17.4%)	0.33
Laboratory test on admission				
White blood cell count (10^3^/mL)	10.2 [3.8–14.2]	6.9 [2.9–15.0]	11.9 [7.5–14.1]	0.17
Platelet counts (10^3^/mL)	159 [74–214]	102 [57–176]	201 [159–306]	*0.001*
Hemoglobin (g/dL)	10.1 [8.8–11.3]	9.4 [8.4–10.5]	11.1 [9.7–12.0]	*0.03*
Prothrombin time (%)	53.4 [37.3–75.0]	42.0 [31.1–56.0]	69.4 [53.1–86.9]	< *0.001*
Lactate (mmol/L)	4.1 [1.7–6.5]	4.6 [3.3–6.6]	2.2 [1.3–6.1]	*0.006*
Septic shock, n (%)	43 (71.7%)	29 (93.5%)	14 (48.3%)	< *0.001*
PMX-HP therapy, n (%)	23 (38.3%)	16 (51.6%)	7 (24.1%)	0.06
**Clinical outcomes**				
In-hospital death, n (%)	18 (30.0%)	14 (45.2%)	4 (13.8%)	*0.01*
Length of ICU stay, day (median, IQR)	3.0 [1.0–8.0]	4.0 [2.0–10.0]	2.0 [1.0–7.0]	0.08
EA level, units (median, IQR)				
T0	0.62 [0.43–0.71]	0.69 [0.67–.077]	0.40 [0.30–0.48]	< *0.001*
T24	0.44 [0.32–0.59]	0.54 [0.38–0.68]	0.35 [0.25–0.45]	< *0.001*
T48	0.39 [0.20–0.62]	0.50 [0.25–0.64]	0.35 [0.18–0.45]	*0.046*
SOFA score (median, IQR)				
T0	6.0 [2.0–12.0]	10.0 [7.0–16.0]	2.0 [1.5–5.5]	< *0.001*
T24	5.0 [1.0–12.0]	8.0 [5.8–14.3]	1.0 [0.0–6.0]	< *0.001*
T48	4.0 [1.0–11.0]	8.0 [4.5–14.0]	1.0 [0.0–5.5]	< *0.001*
T72	4.0 [1.0–9.0]	6.0 [3.0–13.3]	1.0 [0.0–6.5]	*0.001*

*APACHE* Acute physiology and Chronic Health Evaluation, *EA* Endotoxin activity, *ICU* Intensive care unit, *IQR* Inter-quartile range, *PMX-HP* Polymyxin B-hemoperfusion, *SD* Standard deviation, *SOFA* Sequential Organ Failure Assessment.

Significant values are in [italics].

### Effect of PMX-HP on organ dysfunction

**Figure 3:**
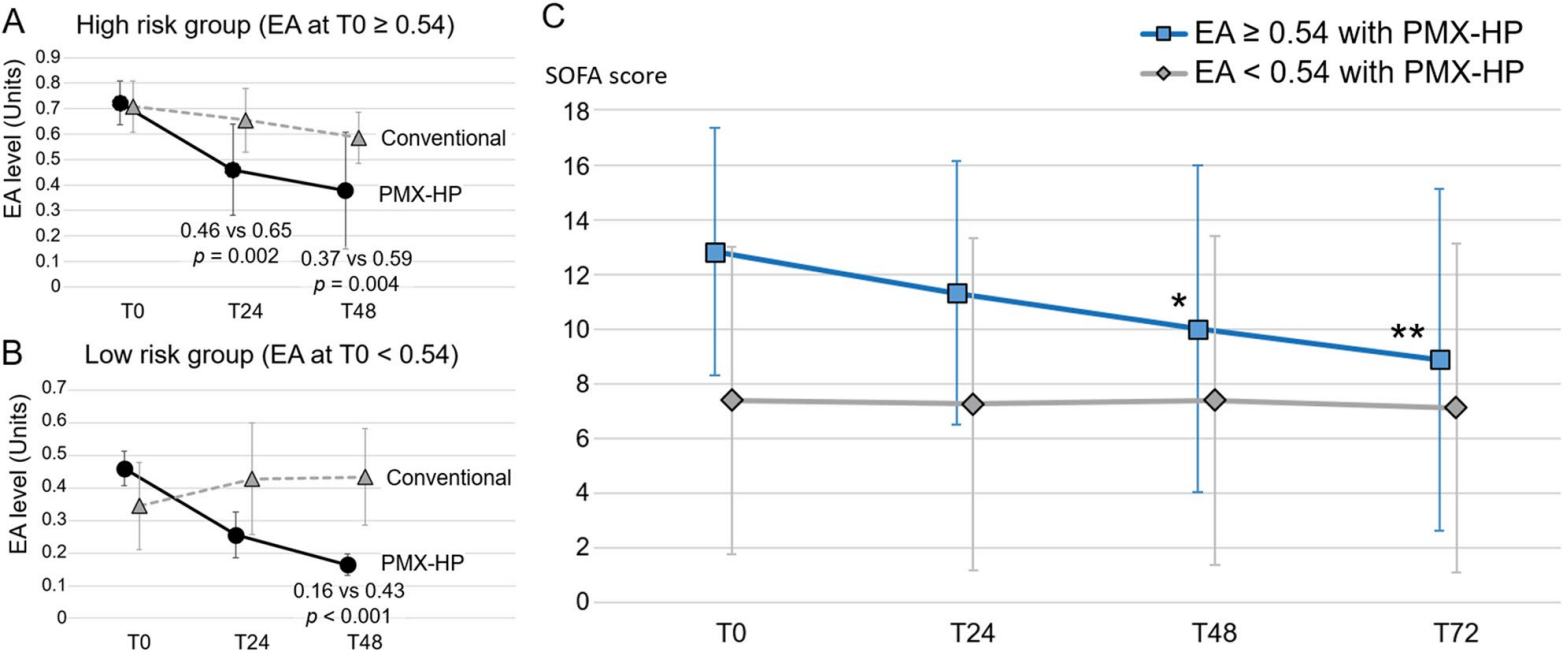
The comparison of changes in EA level with SD by period according to the conductance of PMX-HP or not. With PMX-HP treatment, difference of EA level showed significant time-by-group interactions in both high risk group (**A**, F = 7.56, *p* = 0.003) and low risk group (**B**, F = 10.58, *p* < 0.001). C, changes of SOFA scores with SD in patients who received PMX-HP treatment. The high risk group (blue, EA at T0 ≥ 0.54) showed continuous improvements in SOFA scores, whereas no improvements were revealed in the low risk group (gray, EA at T0 < 0.54). *p < 0.05 vs. baseline (T0); **p < 0.01 vs. baseline (T0). *EA* endotoxin activity, *PMX-HP* polymyxin B-hemoperfusion, *SD* standard deviation, *SOFA* Sequential Organ Failure Assessment, *EA* endotoxin activity *PMX-HP* polymyxin B-hemoperfusion, *SOFA* Sequential Organ Failure Assessment.

Figure 3A,B shows the comparison of changes in EA levels by period according to the administration of PMX-HP. In the high-risk group (EA ≥ 0.54, 31 patients, Fig. 3A), 16 patients (16/31, 51.6%) received PMX-HP and revealed significant decreases in EA levels between T0 and T48 compared to the patients who underwent conventional treatment only (time-by-group interaction, F = 7.56, *p* = 0.003), showing significantly lower EA levels at T24 and T48 with PMX-HP compared to those without PMX-HP (T24: 0.46 vs 0.65, *p* = 0.002, T48: 0.37 vs 0.59, *p* = 0.004). In the low-risk group (EA < 0.54, 29 patients, Fig. 3B), seven patients (7/29, 24.1%) who received PMX-HP, also showed significant decreases in EA levels with time-by-group interaction (F = 10.58, *p* < 0.001). EA level at T48 was significantly low between the two groups (0.16 vs 0.43, *p* < 0.001). In those who underwent PMX-HP treatment, Fig. 3C shows the changes in SOFA scores by period according to the EA risk groups. The patients in the low-risk group receiving PMX-HP showed no improvements in SOFA scores between T0 and T72, whereas patients in the high-risk group receiving PMX-HP showed continuous improvements in SOFA scores during the same period (12.8–8.9, *p* = 0.007).

### Effect of PMX-HP on mortality

In the current study, a total of 14 in-hospital deaths occurred (14/31, 45.2%) in the high-risk group (EA ≥ 0.54), and four in-hospital death occurred (4/29, 13.8%) in the low-risk group (EA < 0.54). In multivariate logistic regression, PMX-HP had a protective effect on in-hospital death in the high-risk group (EA ≥ 0.54, OR: 0.04, 95% CI 0.01–0.71, *p* = 0.03), whereas it had no significant effect on in-hospital death in the low-risk group (EA < 0.54, *p* = 0.22). The SOFA score at T0 was identified as a significant risk factor of in-hospital death in both high-risk patients (EA ≥ 0.54, OR: 1.56, *p* = 0.007) and low-risk patients (EA < 0.54, OR: 0.28, *p* = 0.047) (Table 2).

**Table 2:** Risk factor analysis for in-hospital death in high risk patients (EA ≥ 0.54 at T0) and in low risk patients (EA < 0.54 at T0).

	Univariate analysis		Multivariate analysis	
	OR (95% CI)	*p* value	OR (95% CI)	*p* value
**In high risk patients (EA at T0 ≥ 0.54)**				
PMX-HP treatment	0.53 (0.13–2.20)	*0.18*	0.04 (0.01–0.71)	*0.03*
Age (years)	1.01 (0.94–1.08)	*0.76*		
APACHE II at admission	1.01 (0.93–1.09)	*0.83*		
SOFA score at T0	1.24 (1.03–1.49)	*0.02*	1.56 (1.13–2.16)	*0.007*
Duration of surgery (min)	1.01 (0.99–1.01)	*0.47*		
**In low risk patients (EA at T0 < 0.54)**				
PMX-HP treatment	4.00 (0.45–35.79)	*0.22*		
Age (years)	1.03 (0.94–1.13)	*0.54*		
APACHE II at admission	1.16 (0.99–1.36)	*0.055*		
SOFA score at T0	1.28 (1.00–1.64)	*0.047*	1.28 (1.00–1.64)	*0.047*
Duration of surgery (min)	0.98 (0.96–1.00)	*0.078*		

*APACHE* Acute physiology and Chronic Health Evaluation, *CI* confidence interval, *EA* Endotoxin activity, *OR* Odd ratio, *PMX-HP* Polymyxin B-hemoperfusion, *SOFA* Sequential Organ Failure Assessment.

Significant values are in [italics].

## Discussion

The detection of high EA levels in septic patients may be used as a signal for initiating PMX-HP, a direct endotoxin removal treatment, which is expected to resolve and prevent multi-organ failure induced by septic shock^1^. Our results showed that patients with abdominal sepsis or septic shock experienced significant improvements in EA levels and SOFA scores by receiving additional PMX-HP treatment after infection source-removing surgery. In terms of in-hospital death, however, PMX-HP treatment failed to show a survival benefit (OR: 1.44, *p* = 0.53) in the logistic regression analysis of all participants. Recent randomized trials also reported negative results and it is still controversial whether PMX-HP treatment translates to the improved survival of septic shock patients^14,19–22^. The EUPHAS trial in 2009^14^ reported improved 28-day mortality, hemodynamics, and organ dysfunction from intra-abdominal sepsis in patients where the septic focus was removed surgically. However, the EUPHRATES trial^19^ and ABDOMIX trial^20^ failed to identify benefits in 28-day mortality. The Reasons for these inconsistent results might be explained by the heterogeneity of the patients and the clinical characteristics. First, the EUPHRATES trial included a broad spectrum of infection sites causing sepsis or septic shock^19^. Among these patients, the infection sources were controlled only by antibiotics, which might have a different effect after the same PMX-HP sessions compared to patients whose infection foci were eliminated surgically^14^. Without controlling the infection source, recurrent exposure to endotoxin would exceed the adsorptive capacity of the PMX-HP cycle, causing ongoing organ failure^8,23^. Secondly, with various isolated microorganisms from those patients, the theoretical concept to treat sepsis patients by removing circulating gram-negative endotoxin using PMX-HP treatment might be limited^14,19,20^. Finally, establishing a specific therapeutic target for appropriate timing to initiate PMX-HP could be difficult since the measurement of EA levels was performed in different situations with various clinical conditions. Most previous studies were designed by measuring EA levels in septic shock patients, and enrolled them in PMX-HP treatment when their EA level was 0.6 or greater^1,19^. However, measuring the EA level in a state that has already progressed to septic shock may not be appropriate as a screening tool to predict later progression to septic shock or a predictor of poor prognosis. Besides, the patient who expresses extremely high EA levels in shock status might not have a beneficial effect of PMX-HP if therapy is initiated after the onset of irreversible damages^24^. Romaschin et al.^25^ reported that EA levels exceeding 0.90 might have an endotoxin burden exceeding the adsorptive capacity of the PMX-HP cartridge. Other studies, including our previous study in 2019, EA levels were not measured and failed to identify the survival benefit of PMX-HP treatment. Without the EA levels, the possibility of enrolling the patients who might not benefit from PMX-HP treatment cannot be ruled out.

Novelli et al.^26^ reported the clinical effect by decreased SOFA scores and improved hemodynamics of PMX-HP in post-abdominal surgery patients with high EA levels (≥ 0.6) which were measured when the patient showed signs of systemic inflammatory response syndrome. However, clinical implication of that study might be difficult since they included post-transplant patients with immunosuppressant, and failed to identify the difference of survival against the conventional treatment group.

These limitations necessitate identifying the most favorable patient population for effective PMX-HP treatment. Moreover, PMX-HP treatment should have a clear survival benefit since it is a high-cost therapy requiring complex procedures including a Toraymyxin cartridge in the CRRT equipment and central vascular access. The results of our study revealed that the high-risk group (EA ≥ 0.54) experienced significant improvements in SOFA scores and decreased in-hospital deaths, however, low-risk patients (EA < 0.54) had no benefit to organ dysfunction and mortality from PMX-HP treatment, as shown in Table 2 and Fig. 3-C. These results could be explained by plotting the probability of in-hospital death with PMX-HP versus the conventional group across the EA levels at baseline, in which the two curves intersected between 0.5 and 0.6, indicating that PMX-HP had a survival benefit in patients with higher EA levels. This result might be supported by the theoretical concept that elevated levels of EA would be most likely to respond to PMX-HP^19,26^.

Identifying the appropriate timing for PMX-HP treatment is also important. The present study measured EA levels immediately after ICU admission to detect septic shock. The results of this study revealed patients with high EA levels (> 0.54) at acute period may experience more severe clinical conditions including septic shock and higher probability of mortality which are similar results with the other studies^1,2,7^. Within these high risk patients, prompt PMX-HP treatment was performed before irreversible organ damages occurs, and showed significant decreasing EA levels with survival benefit. Reducing EA level at the early phase might have improved clinical outcomes which findings are in accordance with Klein et al., that organ dysfunction was associated with the total burden of endotoxin within the first 72-h period after ICU admission^23^. Therefore, it might be useful to decide the early administration of PMX-HP treatment if EA level is high in the acute admission period.

Despite the interesting results, our results should be interpreted with cautions due to the study limitations. First, the decision to use PMX-HP treatment was not standardized due to the nature of the observational cohort study. Also, unmeasured confounding factors including the socioeconomic status and medical insurance status of the patient could have influenced the treatment decision to use PMX-HP, which is a costly treatment. Secondly, despite the highly controlled patient enrolment, there was a broad spectrum of clinical characteristics with different disease severity within the patients who received PMX-HP treatment, and the dose, duration, and number of cycles of PMX-HP treatment were applied without considering these individual differences. Thirdly, although we used empirical antibiotic agents according to our institution’s policy, such as meropenem, piperacillin/tazobactam, or vancomycin, which are known to have coverage against major strains of intraabdominal infection, we could not analyze the adequacy of empirical antimicrobial therapy. This is because there were no accurate data on empirical antibacterial agent for each patient due to the unavoidable limitations of the retrospective analysis. Fourthly, the number of patients and death events were too small to suggest a specific cut-off EA value by performing a ROC analysis for ICU-, 7-day, 28-day or In-hospital death. Finally, we enrolled only the patients whose infectious source was successfully controlled. Therefore, the clinical implication should be different in non-successful source controlled patients and medical sepsis population in which the patients are repeatedly exposed to endotoxin. Further studies are needed for generalization of the treatment strategy in a detailed protocol for using PMX-HP therapy in patients who failed to remove the infection source completely and when EA level remains high.

Despite these shortcomings, our study has several advantages that differentiate it from previous studies. To the best of our knowledge, this was the first study that included a large number of data in a homogeneous subset of patients showing the serial trend in endotoxin activity after surgery. We believe that our results can suggest detailed guidelines under which to perform PMX-HP treatment (EA ≥ 0.54) on an infectious source-controlled patient with abdominal sepsis, which can generate survival benefit and recovery from organ dysfunction. Additionally, an EA cutoff level 0.54 could facilitate decision-making on performing PMX-HP therapy in marginally ill patients with EA levels between 0.4 and 0.6, which is regarded as an intermediate-range^26,27^. Most previous studies were designed by enrolling the patients in PMX-HP treatment when their EA level was 0.6 or greater. The MEDIC study, however, revealed that patients who shows intermediate EA level, in-hospital mortality might be high as in the high EA group (≥ 0.6)^7^. Therefore, our cut-off value of 0.54 might be useful to consider the PMX-HP treatment in this group of patients. A large-scale randomized prospective study should be conducted to confirm the results of the current study in the near future.

In conclusion, EA level significantly decreased after PMX-HP treatment and it may represent a therapeutic option to improve organ impairment and in-hospital death in septic shock patients with EA levels exceeding 0.54. In patients with low EA levels (< 0.54), PMX-HP treatment seemed to have no benefit in improving organ dysfunction and survival.

## Supplementary Information

Supplementary Table S1.
