# Supplementary material for: Impact of polymyxin B hemoperfusion therapy on high endotoxin activity level patients after successful infection source control: a prospective cohort study
Source: Sci Rep. 2021 Dec 16;11:24132. doi: 10.1038/s41598-021-03055-8 (PMC8677752; doi:10.1038/s41598-021-03055-8)
Supplement: Supplementary file 1 — Supplementary Table S1. [file 41598_2021_3055_MOESM1_ESM.docx]

**Supplementary**

Table S1. The indications and contraindications for PMX-HP

| **Indications** |
| --- |
| - Age 18 years or older |
| - Worsening or sustained septic shock despite proper antibiotic treatment and surgery or intervention for effective source control |
| - Clinical manifestations of sepsis or septic shock associated with intraabdominal inflammation with a Sequential Organ Failure Assessment (SOFA) score more than 2 |
| - The cause of sepsis was a gram-negative infection confirmed through multiple site cultures or clinically suspected |
| - The need to administer a vasopressor in high doses within 12 hours after diagnosis |
| **Contraindications** |
| - Age under 18 years |
| - Previous history of hypersensitivity to PMX-HP |
| - Failure of source control due to the worsening condition of the patient |
| - Severe thrombocytopenia (platelet count < 30000$\times$10^9^/L) |
| - The use of an immunosuppressive drug |
| - Uncontrolled active bleeding within 24 hours after surgery or intervention |
| - Hematologic malignancy |
| - Severe leukocytopenia (leucocyte count of < 500 µl/L) |
| - Pregnancy |
